# Target switching in 2D and 3D visual foraging reveals trade-offs between mental effort, travelling distance and movement speed

**DOI:** 10.1371/journal.pone.0342298

**Published:** 2026-02-12

**Authors:** Emre Orun, Robin J. Green, Carlo De Lillo

**Affiliations:** School of Psychology and Vision Sciences, University of Leicester, Leicester, United Kingdom; RIKEN CBS: RIKEN Noshinkei Kagaku Kenkyu Center, JAPAN

## Abstract

Many everyday activities entail searching for multiple desired items and can be conceptualised as foraging. They require mental effort to keep each item in memory, while searching. An instantiation of such activities in experimental psychology is visual foraging, where people search for multiple target categories among distractors. In visual foraging, high frequencies of switching between selections of two different target categories suggest the use of mentally effortful strategies, which require sustained simultaneous use of working memory templates for each of them. As humans often strive to reduce mental effort in cognitive tasks, a key theoretical issue is the characterisation of what induces people to spontaneously increase the frequency of switching between target categories. In five experiments, we systematically manipulated variables in instances of foraging in 2D and 3D virtual reality environments to assess changes in target switching frequency and its determinants. Experiment 1 showed that humans switch more when foraging in large immersive navigational environments, than in visual arrays seen from a bird’s eye view. Further experiments clarified that: 1) inter-target distance and movement speed are the critical determinants of strategy changes in a 2D small-scale environment; 2) distance affects the frequency of switching more than speed, in a 3D navigational environment. These results indicate that people spontaneously choose to endure mental costs to meet the demands of different naturalistic instances of foraging. They support theories postulating a flexible use of working memory templates.

## Introduction

Search, and its instantiation in foraging, is an almost ubiquitous behavioural and cognitive process [1]. It can support the exploration of alternatives to find desirable visual targets and foraging for food resource. Experimental psychology has most often focused on cognitive (attentional and working memory) underpinnings of visual search for visible targets presented on a computer screen in laboratory settings [2]. Conversely, field studies of foraging for food in larger scale environments often test predictions of theoretical models of optimisation of energy intake and expenditure [3,4]. Because energy intake and expenditure are difficult to measure, typically time spent foraging and distance travelled to collect food are used as their proxies [5,6].

Some authors advocate the analogy of visual search and foraging behaviour and their possible common evolutionary origin [7,8]. However, it has been pointed out that visual search and foraging differ both in the number of targets that need to be found [9] and in the scale of the search environment. Gilchrist et al. [10] suggested that foraging in large-scale environments differ from visual search in small-scale displays because of its greater reliance on cognitive resources (memory) required to avoid costly (in terms of time and energy) rechecks of stimuli at different locations.

In the present paper, we take the stance that processes analogous to visual search take place in particular instances of foraging. For example, a forager may engage in visual search when scanning for prey on a flat surface and has to visually identify targets (e.g., think of a pigeon searching for grains among items of dirt on the ground, or a human who separates lentils from stones in a sieve before cooking). However, in order to locate profitable resource patches, a forager will need to navigate to explore large areas. Terrestrial organisms, such as humans, do not have the option to visually scan these areas with a bird’s eye view and processes analogous to those described by Gilchrist et al. [10] will have to take place. As such, there may be a tension between the requirements of these two types of foraging and the different trade-off between cognitive and energy costs that they imply.

Studies of search in large-scale semi-naturalistic foraging tasks have suggested that humans [11–13], as well as other primates [14–16], develop search strategies to minimise the working memory load imposed by having to keep track of visited locations. However, in these studies, behavioural strategies allowing the minimisation of cognitive costs often coincide with those that would allow reducing movement length [11]. Thus, they fail to unambiguously determine the trade-offs which underpin changes in search strategies.

Adapting visual search paradigms to make them mimic foraging for multiple target categories [17,18] can provide the opportunity to do so, because searches requiring shorter movements in visual foraging often impose a higher cognitive load. Kristjánsson et al. [18] used a visual foraging task where participants had to search for targets belonging to two colour categories (e.g., red and green disks) within a small two-dimensional display containing both targets and distractors (e.g., yellow and blue disks) presented on an iPad. In a more challenging condition, targets (e.g., red squares and green disks) and distractors (e.g., green squares and red disks) were defined by a conjunction of features. As explained in more detail below, the authors used the frequency of behavioural switching across target categories during search (e.g., consecutively selecting a red and a green disk) to measure the extent to which participants engaged in more or less cognitively demanding strategies.

A key manipulation in the study by Kristjánsson et al. [18] was the time allocated to participants to complete search, with a condition without time limits compared to conditions with time limits of 5, 10, 15 seconds, respectively. Kristjánsson et al. [18] showed that imposing time limits on people’s search make them switch more frequently between target categories. They suggested that such increase in switching frequency is evidence of more flexible use of working memory templates than predicted by models which assume that only one working memory template can be activated at any particular point in time during search. In fact, according to Kristjánsson et al. [18] high rates of switching require high-level concentration to enable multiple working memory templates to guide search at any one time.

In the study by Kristjánsson et al. [18], time limits were not considered as inherent to particular instances of foraging. Rather their length was manipulated by the experimenters to test if providing less time encouraged people to switch more. A crucial interpretation provided by Kristjánsson et al. [18] of their results is that time limits encourage participants to engage in more effortful strategies which allow them to select more targets in the time available. In other words, bursts of high concentration which allow keeping more templates active at the same time would help minimise movement costs (as some targets would need to be skipped and selected by returning to them at a later stage during search, if switches between target types are minimised). Added search time caused by these additional movements would result in fewer items selected when the available time elapses. According to the authors, the incentive for using more mentally effortful strategies is provided by the reduced likelihood of completing an exhaustive search of all the targets in trials with short time limits. By contrast, when there is no time limit, there would be no incentive to use mentally effortful strategies because the forager will eventually search the whole display and find all the targets.

The hypothesis of a flexible use of working memory templates, as evidenced by switching frequency or related measures, has found support in several studies. For example, Thornton et al. [19] found that manipulating the speed of a metronome that participants had to use to guide their foraging affected the rate of switching between target types. Similarly, inducing a sense of danger, by requiring participants to move their cursor away from images of “predators” during foraging, increases their switch rate [20], as does the increase in complexity of the search task [17]. Many of such studies take an increase of switch rate as an indication of foragers engaging in more cognitively costly strategies.

People normally strive to avoid mental effort and even endure physical pain to do so [21]. Therefore, it is of considerable theoretical interest to determine the conditions that induce people to spontaneously engage in mentally effortful strategies. Trade-offs between physical and mental effort have been shown in studies which have used a hand dynamometer to manipulate the physical effort required to search for targets and their numerosity to manipulate the attentional effort required to search [21,22]. Studies which have dissociated head and eye movement in visual search for letters have found more memory involvement when head movements (considered more energetically demanding than just eye movements) were involved [23]. Varying the resistance of movements using a robotic handle can bias manual search (moving a cursor to different location to see if it contains the target) but not visual search [24]. Trade-offs between energy expended in movement (simulated by giving participants a digital energy budget expended at different rates for uphill or downhill/flat terrain movements) or in cognitive activities such engaging in perception and movement planning (assumed to occur during movement pauses) have been shown in virtual foraging studies [25]. Thus, several studies suggest a complex, and often difficult to disentangle, relationship between mental effort, physical effort and search behaviour.

The aim of our study was to use visual foraging in different types of set-ups as a way of clarifying further the trade-off between movement costs and cognitive costs in search. Our hypothesis was that participants should switch more, and thus show to engage in more effortful strategies, when implicit costs, imposed by foraging type and spatial scale of the foraging environment, make it worthwhile. We used a similar approach to that of Kristjánsson et al. [18], to assess if two naturalistic occurrences of foraging resembling, respectively, foraging for items in a small-scale space when a bird’s eye view of the search array is permitted and in larger scales where this cannot happen, produce changes in switching frequency. Crucially, and differently from Kristjánsson et al. [18], by not setting specific time limits, our experiments aimed to clarify if the implicit demands provided by different types of foraging are sufficient to induce an increase in the number of switches between target categories, even when participants have unlimited time to complete an exhaustive search of all the targets.

In our first experiment, we used a task similar to that used by Kristjánsson et al. [18], and we presented it either on a 2D monitor offering a bird’s eye view of the search space or in a 3D virtual reality environment requiring navigation. There are numerous studies examining the effects of display size in 2D contexts [26–28]. Virtual reality studies are rarer. Kristjánsson et al. [29] used a virtual reality task to determine if moving targets, that participants were required to shoot with a laser beam, induced more switches that static ones. However, this manipulation did not produce significant differences in switching patterns. To our knowledge, there are no studies comparing switching behaviour in an immersive 3D navigational environment and a 2D display seen with a bird’s eye view. Based on the arguments outlined above, we considered that this manipulation was a particularly ecologically relevant one to perform.

We predicted that the additional movement costs associated with searching within a 3D navigational environment for one category of targets at any one time would offset the cognitive costs required by holding more templates in visual working memory and switching frequently between them. We also predicted that differences in the switch frequency observed in the two environments would be particularly noticeable when targets are defined by a conjunction of two features. This is because the complexity of conjunction templates will add to the cognitive costs implied in holding them simultaneously in visual working memory to allow frequent switches [17]. Thus, foragers would only take on the extra cognitive costs when they are offset by the additional movement costs implicitly imposed by foraging in a large-scale 3D environment. By contrast, switching rate may be high across environments when targets are defined by a single feature, due to relatively low cognitive costs of holding feature templates simultaneously in working memory for sustained periods.

Having tested these predictions in our first experiment, we aimed to determine the specific role of movement speed and distance in further experiments where these variables were manipulated within 2D and 3D search spaces, respectively.

## Experiment 1

Experiment 1 aimed to determine if the relative demands of foraging in a 2D small-scale environment and 3D large-scale environment affected the number of switches between target categories performed by the participants during search. Following previous studies [18], Experiment 1 also aimed to assess the extent to which searching for targets defined by single features or conjunctions of features affected switching frequency, and if this manipulation interacted with the type of search environment.

### Method

#### Data availability.

The data set for this and all the other experiments, as well as videos showing examples of trials in 3D environments for this and other experiments reported in this article, have been made freely available online from the Open Science Framework https://osf.io/pc5gt/.

#### Ethical approval and participant recruitment.

Experiment one and all the subsequent experiments reported in this paper were approved by the University of Leicester’s Psychology Research Ethics Committee (approval numbers: 22311-eo126-ls:neuroscience,psychology&behaviour and 27750-eo126-ls:neuroscience,psychology&behaviour) and in accordance to the British Psychological Society’s Code of Human Research Ethics. Participants were provided for each experiment with an information sheet and provided written informed consent through signing a consent form. The recruitment period for the experiments in this paper was from 07/10/2019–01/08/2022.

#### Participants.

For this and subsequent experiments (as all experiments reported here used the same 2 x 2 repeated measures design), an a priori power analysis was performed using G*Power 3.1.9.7 [30,31]. The power analysis showed that at least 24 participants were needed to detect significant effects based on Cohen’s [32] medium effect size (*f* = 0.25), a power of 0.8 using the “ANOVA: repeated measures within factors” procedure. We used 0.8 as the power determinant and medium effect size as this study is not based on previously established norm effect sizes in the field. This is a common convention that many studies base a priori power analysis on, despite known flaws [33,34]. As our experiment used immersive virtual reality, which knowingly induces motion sickness, we over recruited to compensate for possible attrition due to participants withdrawing from the experiment. Participants provided informed consent (written) to partake in the study. As all participants were adults, they were able to provide consent for themselves.

Thus, sixty-four (10 males, 54 females) students from the School of Psychology and Vision Sciences at the University of Leicester took part. They received course credits for taking part. The age range was 18–20 years (*M* = 18.53 years, *SD* = 0.64 years). All participants had either normal or corrected-to-normal vision. Fifty-seven participants were right-handed and seven were left-handed.

#### Design.

We used an adapted version of the visual foraging paradigm by Kristjánsson et al. [18]. A 2 (Environment: 2D small-scale, 3D large-scale) x 2 (Stimulus Type: feature, conjunction) repeated measures experiment was conducted. Hence, four conditions were administered: 1) 2D small-scale feature; 2) 2D small-scale conjunction; 3) 3D large-scale feature, and 4) 3D large-scale conjunction. The order of presentation of the different conditions was counterbalanced according to a Latin Square design. The dependent variable used for the experiment was the frequencies of switches performed by the participants, whereby a switch was defined as the selection of a target type different from the target type of the previous selection.

#### The general foraging task.

Both 2D small-scale and 3D large-scale displays in Experiment 1 required participants to forage within an array to select two different targets. Two different target types were available (e.g., red circles and green circles). So, there would be only one switch when participants select all the available red circles before selecting all green circles. By contrast, switching between targets regularly would result in a high frequency of switches.

In the feature condition, a feature refers to a single property (colour) distinguishing each stimulus. The targets differed from non-targets on the basis of their colour exclusively. In the conjunction condition, stimuli had two properties (colour and shape), with distractor stimuli sharing one property with target stimuli.

Each condition consisted of 20 trials. In each trial, 40 targets and 40 distractors were presented on a computer monitor. Targets and distractors were randomly distributed on a jittered grid, which was created by adding a random vertical and horizontal offset to create a less uniform appearance than a square matrix without jittering. Participants were informed regarding which coloured/shaped stimuli were targets and which were distractors, e.g., a red sphere and a green sphere as the two targets and a blue sphere and a yellow sphere as distractors for a feature condition or a red cube and a green sphere as targets and a red sphere and a green cube as distractors for a conjunction condition. For half of the participants, target and distractor stimuli were reversed for counterbalancing, e.g., blue/yellow targets and red/green distractors, based on Kristjánsson et al. [18]. Participants were also told that selecting a distractor would result in an error, which would end the current trial and trigger the presentation of the following trial. Targets disappeared once selected. Each trial was successfully completed once no targets were left within the environment. To account for order/practice effects between colours, dimensions, and type of condition, eight separate sequences of conditions were used for counterbalancing. An example of one sequence would be: 3D large-scale feature foraging with red/green sphere targets, 3D large-scale conjunction foraging, with red cube/green sphere targets, 2D small-scale feature foraging with red/green circle targets, and then 2D small-scale conjunction foraging with red squares/green circle targets. As participants were not explicitly instructed to switch between targets in the experiment, any switching would be performed spontaneously by the participant.

Prior to the administration of each of the 3D conditions, participants completed two practice trials to familiarise themselves with the task and the controls. Practice trials that preceded the feature conditions displayed a 2 x 4 array of poles, four of which were surmounted by four white spheres and four by grey spheres. Participants had to select all grey poles. For practice trials that preceded the conjunction conditions, the shapes surmounting the eight poles were assigned as follows: 2 x white spheres; 2 x grey spheres; 2 x white cubes and 2 x grey cubes. Participants were asked to select all grey cubes and white spheres.

Details pertaining to the specific displays and the environmental differences regarding how the task was conducted are presented below.

#### The 2D small-scale foraging environment display.

**Fig 1:**
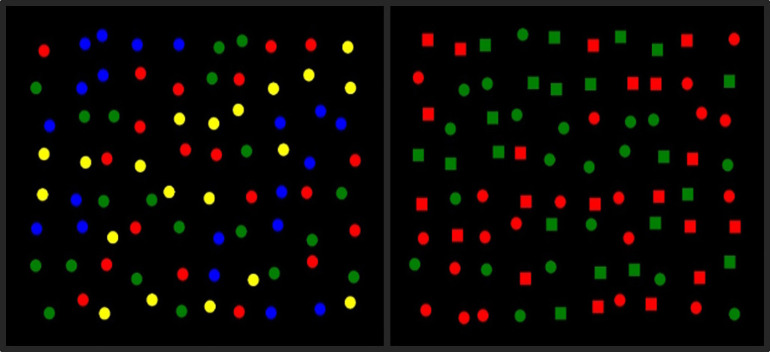
2D small-scale foraging display for feature and conjunction conditions. An example of a 2D small-scale display feature trial (left), where targets and distractors are single colour features (red circle, green circle, blue circle, yellow circle), and a 2D small-scale display conjunction trial (right) where targets are defined by colour and shape (red square, red circle, green square, green circle). Correct selections made the target disappear whereas incorrect selections immediately ended the trial.

E-Prime 2.0 [35] was used to program the 2D small-scale environment condition, which was displayed on a touch-sensitive PC monitor – Elo TouchSystems ET1925L-8UWA-1 (37.6 cm wide x 30.1 cm high). The array used for the 2D small-scale display comprised 80 images arranged as a grid. The images were jittered by adding a random vertical and horizontal offset to create a less uniform appearance (see Fig 1). The array was presented within a screen area of 1280 x 1024 pixels. The array could not extend beyond a margin of 107 pixels on the left and right (about 8.4% of screen width) and 93 pixels at the top and bottom (about 9.1% of screen height). Each image had a width of 40 pixels (about 3.1% of screen width and 3.9% of screen height). Jittering involved the random displacement of images from their grid positions in the x and y directions. Images were prevented from touching or overlapping with each other by setting the maximum jitter distance to 80% of half the distance between adjacent images in the grid (about 30 pixels). The images were assigned to array positions according to a fixed (not randomised) sequence. Red, Green, and Blue (RGB) intensity values of the stimuli were: red (255, 0, 0), green (0, 128, 0), blue (0, 0, 255), yellow (255, 255, 0). The 2D small-scale condition presented circle/square stimuli on a black background (see Fig 1).

#### The 3D large-scale foraging environment display.

**Fig 2:**
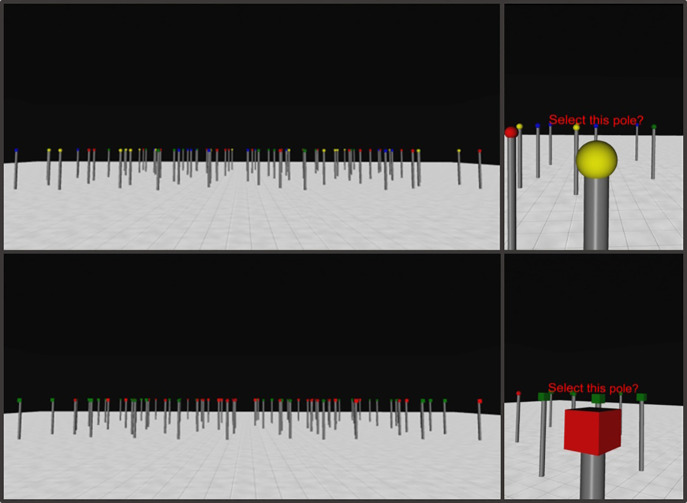
3D large-scale foraging display for feature and conjunction conditions of Experiment 1. An example of a 3D large-scale feature foraging trial (top) where targets and distractors are defined by colour only, and a 3D conjunction foraging trial (bottom) where targets are distinguished by colour and shape. When participants trespassed a predefined proximity threshold, they had the option to select the stimulus. Examples of viewpoints within the proximity area where selection of the stimuli was possible are shown in the panels on the right.

Vizard 5 [36] was used to create the 3D large-scale environment, which was viewed on an ASUS VG278HE PC monitor (60 cm wide x 34 cm high), via 3D glasses (NVIDIA 3D Vision 2). A PlayStation 4 controller enabled navigation. For the 3D environment, the array consisted of 80 poles surmounted by 3D objects arranged as a grid and jittered, similarly to what was done for the 2D small-scale display. The array was situated in the centre of a virtual room (see Fig 2). The size of the virtual room was set to ensure that the participant could see the whole of the array from a starting position close to one wall of the room. The virtual room dimensions were measured in virtual metres (vm), which were scaled with reference to the height of the participant wearing the headset, ensuring that the ratio of actual metres versus vm would be about 1:1. The size of the virtual room was 49.18 vm wide by 49.18 vm long by 7.26 vm high. The distance between poles was 2 vm.

Jittering involved the random displacement of poles from their grid positions in the x and y directions. Poles were prevented from touching or overlapping by setting the maximum jitter distance to 80% of half the distance between adjacent poles in the grid (0.736 vm for the sparse array and 0.336 for the dense array). After the positions of the poles in the array were determined, the poles (each with a colour and shape) were assigned to these positions according to a randomised sequence. RGB values of the stimuli were: red (255, 0, 0), green (0, 128, 0), blue (0, 0, 255), yellow (255, 255, 0). The 3D condition required the participant to navigate within a 3D environment (the virtual room described above) by operating a joystick on a PlayStation 4 controller with their thumb. The controller allowed them to move forward, backwards and change their heading direction while selecting spherical and/or cubical target stimuli (see Fig 2).

Thus, the viewpoint experienced by the participant changed depending on their heading direction within the virtual environment. Participants were allowed to move across the whole surface of the floor of the virtual room. Selecting a target involved moving towards the pole within the virtual space and clicking a trigger button of the controller (R1), once within a predefined proximity of the stimulus. This predefined proximity was set as 0.45 vm from the stimulus. When participants entered this proximity zone a text prompt appeared above the stimulus asking if participants wanted to select it. Clicking the R1 button at this point recorded the selection. Clicking outside of this proximity zone had no effect and was not recorded.

### Results

Following Kristjánsson et al. [9], before the analyses, the data were cleaned up by deleting all trials where a distractor was selected. This resulted in 1183 trials being removed, and 3937 trials included in the data for analysis, i.e., 38.3% of trials were removed. In more detail, for the 2D Feature condition we removed 66 trials (5.16%), 137 trials (10.7%) in the 2D Conjunction condition, 496 trials (38.75%) in the 3D Feature condition, and 484 trials (37.81%) in the 3D Conjunction condition.

**Fig 3:**
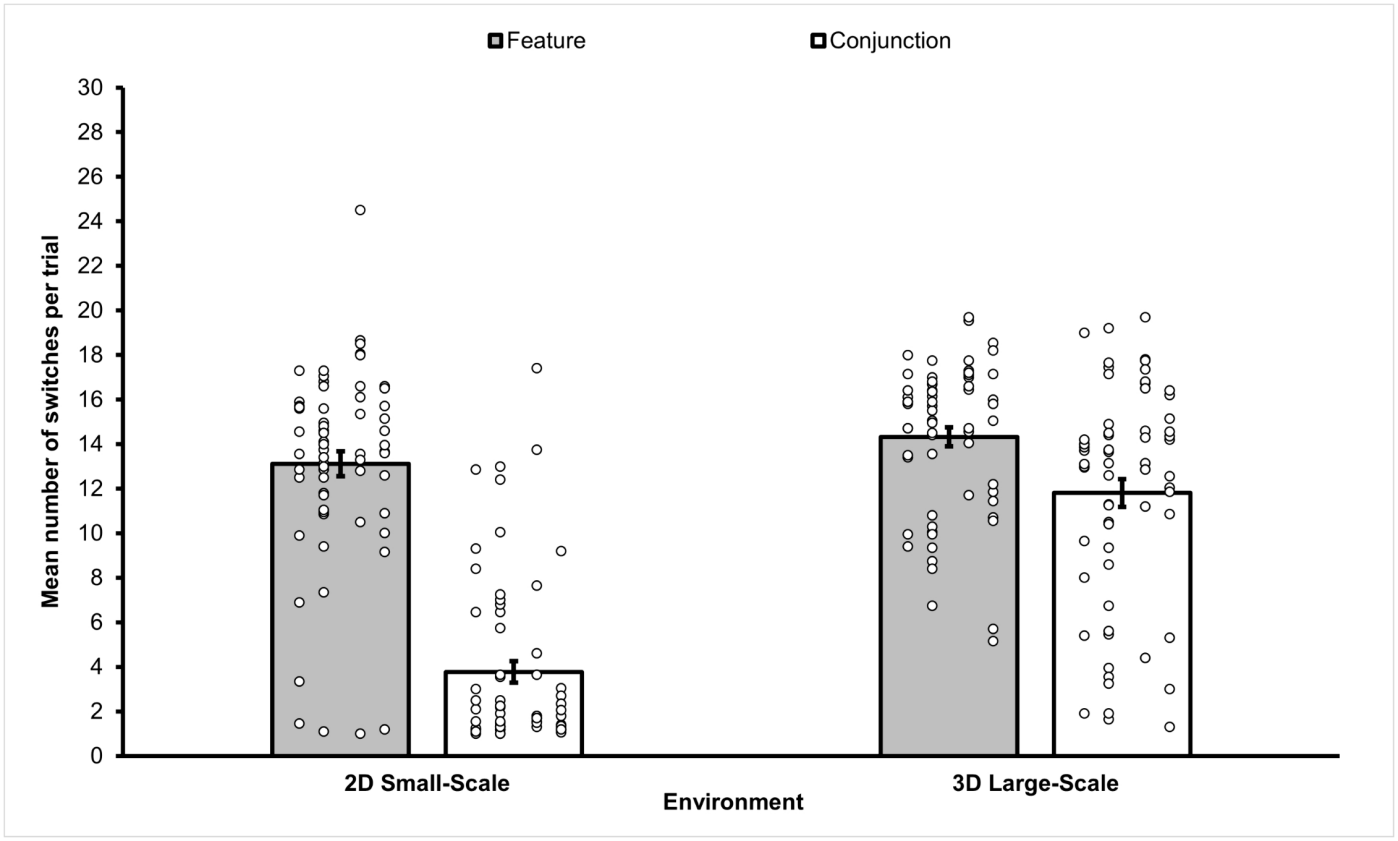
Mean frequency of target switches observed in the different sub-conditions of Experiment 1. Switching frequency per sub-condition showing that overall 3D large-scale conditions elicited more switches than 2D small-scale conditions and feature conditions statistically significantly induced more switches than the conjunction conditions. The figure also illustrates a statistically significant larger difference between the conjunction conditions of the two environments compared to the difference between the feature conditions of the two environments. Error bars represent a standard error of the mean. Small circles represent individual scores.

The mean frequency of switches observed in the four different conditions featured in Experiment 1 is depicted in Fig 3.

For this and all other subsequent experiments, statistical data analyses were carried out using JASP Version 0.18.3 [37].

We ran a 2 (Environment: 2D small-scale, 3D large-scale) x 2 (Stimulus Type: feature, conjunction) repeated measures ANOVA on the frequency of switching to determine if switching behaviour was affected by Environment and Stimulus Type. The results supported the hypothesis of 3D large-scale conditions yielding a higher mean frequency of switches (see Fig 3). In fact, a main effect of Environment was observed (*F*(1,63) = 75.185, *p* < .001, *η*_*p*_^2^ = .54), with a higher mean frequency of switches observed in the 3D large-scale environment (*M* = 13.07, *SEM* = .452, 95% *CI* [7.57, 9.31]) compared to the 2D small-scale environment (*M* = 8.44, *SEM* = .433, 95% *CI* [12.17, 13.70]). A main effect of Stimulus Type emerged also (*F*(1,63) = 231.063, *p* < .001, *η*_*p*_^2^ = .79), with a higher mean frequency of switches observed in the feature condition (*M* = 13.72, *SEM* = .37, 95% *CI* [12.98, 14.46]) compared to the conjunction condition (*M* = 7.79, *SEM* = .34, 95% *CI* [6.92, 8.66]). The interaction between Environment and Stimulus Type was also statistically significant (*F*(1,63) = 68.696, *p* < .001, *η*_*p*_^2^ = .52). Paired sample t-tests revealed that there was a statistically significant difference in mean switches between feature and conjunctions conditions in both 3D large-scale (*t*(63) = 4.485, *p* < .001) and 2D small-scale conditions (*t*(63) = 16.366, *p* < .001). As for comparisons be*t*ween stimulus type in each of *t*he two environments, the mean frequency of switches in the conjunction condition of the 3D large-scale environment was significantly higher than in the 2D small-scale environment (*t*(63) = 11.604, *p* < .001). However, the comparison of the mean frequency of switches for the fea*t*ure conditions of the two environments proved slightly above the threshold of statistical significance (*t*(63) = 1.868, *p* = .066).

An analysis of error frequency (i.e., number of trials removed in the different conditions due to a distractor being selected) was carried out to provide an indication of the relative difficulty of each condition. A 2 (Environment: 2D small-scale, 3D large-scale) x 2 (Stimulus Type: feature, conjunction) repeated measures ANOVA revealed that 3D conditions resulted in a significantly higher frequency of errors (*M* = 7.656, *SEM* = .418, 95% *CI* [0.636, 2.536]) than 2D conditions (*M* = 1.586, *SEM* = .418, 95% *CI* [0.636, 2.536]), (*F*(1,63) = 144.818, *p* < .001, *η*_*p*_*²* = .607). By contrast, there was no statistically significant difference in errors in Conjunction (*M* = 4.852, *SEM* = .354, 95% *CI* [4.043, 5.661]), compared to Feature stimuli conditions (*M* = 4.391, *SEM* = .354, 95% *CI* [3.582, 5.200]), (*F*(1, 63), = 3.847, *p* = .054, *η*_*p*_*²* = .058). The interaction between environment and stimulus type was statistically significant (*F*(1, 63), = 7.173, *p* = .009, *η*_*p*_*²* = .102). Post hoc tests revealed statistically significant differences between each condition (lowest significant comparison *p* = .003) except for the comparison of the 3D Feature and 3D Conjunction (*p* = .579).

Finally, to assess the extent to which switching more was associated with a reduction of the length of the movement path used to complete a trial (where no errors were performed) we carried out Pearson’s R correlations between the frequency of switching and path length. There was a statistically significant negative correlation between number of switches and path length in 2D feature condition (*r*(62) = −.277, *p* = .027), in 3D feature condition (*r*(62) = −.449, *p* < .001) and 3D conjunction condition (*r*(62) = −.726, *p* < .001). However, the relationship between switches and path length was not statistically significant in the 2D conjunction condition (*r*(62) = −.058, *p* = .648).

### Discussion

Experiment 1 assessed the effects of the foraging environment and stimulus type on the frequency of switches between targets. This experiment revealed that a 3D (large-scale) environment induced more target switches than a 2D (small-scale) environment, with feature conditions producing more switches than conjunction conditions. In particular, a 3D large-scale feature foraging condition yielded the highest frequency of switches and 2D small-scale conjunction condition resulted in the lowest number of switches. It is worth noting the large difference between switching behaviour in two environments when conjunction stimuli were used.

The results of Experiment 1 allow us to add new and important findings to those obtained by the pioneering work of Kristjánsson et al. [18], which suggested that WM is flexible enough to allow the use of multiple search templates simultaneously, as a consequence of the task demands generated by imposing explicit time limits on a foraging task.

In Experiment 1 we tested the prediction that the implicit demands of different instantiations of foraging can alter the trade-off between cognitive costs and costs associated with search in terms of energy and time spent searching. Our results suggest that a large-scale navigational environment creates an incentive for people to engage in more costly cognitive strategies, as evinced by the increase in frequency of switches between targets. The difference is particularly evident when the conjunction conditions of the two environments are compared. Because of the load imposed by holding each complex template in working memory, the conjunction condition is the one where people default to use mostly a single template in a 2D environment. Thus, it seems that the task demands of foraging in a visual environment observed from a bird’s eye viewpoint do not add enough extra-cognitive costs to encourage people to switch to more cognitively costly strategies. By contrast, single feature conditions, where templates do not impose a large cognitive load, yield a relatively large frequency of switches in both environments.

Thus, as we hypothesised, instantiations of foraging in environments of different sizes can affect the switching behaviour, even without the explicit imposition of time limits by the experimenter. We can conjecture that there is a trade-off between cognitive and movement (i.e., time, distance, and/or energy) costs demonstrated here, whereby more switches entail higher cognitive costs and lower movement costs, whereas fewer switches entail higher movement costs and lower cognitive costs. If that is the case, our experiment suggests that a higher frequency of switching was spontaneously deployed to reduce the movement costs implicitly imposed by the large-scale navigational environment. For example, if a participant selected a red sphere in the 3D large-scale environment, then opted to select another distant red sphere rather than a nearby green sphere, this would be more costly in terms of length of movement than an equivalent transition between the same two stimuli in the 2D small-scale environment. In fact, there was no significant correlation between switching and path length in the 2D conjunction condition (possibly due to the small number of switches observed in that condition), whereas a significant negative correlation between these two variables in all other conditions reinforces the intuitive notion that switching helps reduce the length of the movement required to perform an exhaustive search for all the targets, in completed trials.

The analysis of error frequencies provides an indication of the relative difficulty of each condition. It can be useful in view of a possible confound of the role of difficulty in identifying the targets and the costs of moving for longer distances in inducing a higher frequency of switches. In fact, it could be argued that in more difficult conditions, people would need to mostly focus attention on one target at any one time, and this would result in fewer switches. Our results suggest that this may not be the case here as participants switched more when foraging the large 3D condition environments, which were also where more errors occurred. This suggests that, in this experiment, the incentive to switch more was provided by the reduction of distance travelled when more switches were performed. Also, conjunction foraging did not prove more difficult than feature foraging, but a significant interaction environment and stimulus type required to be disentangled with post-hoc tests. They showed a significant difference between stimulus type in the 2D small-scale but not the 3D large-scale environment. This pattern of results also suggests a dissociation between switch frequency and difficulty.

The frequency of errors can provide an indication of relative difficulty. Nonetheless, according to our hypotheses it should be the frequency of switching in correct trials to provide the clearest demonstration of the trade-offs between cognitive and movement costs in foraging.

## Experiment 2

The results from Experiment 1 suggest that foraging in a large 3D navigational environment determines an increase in the frequency of switches between multiple target types, compared to foraging in a 2D small-scale environment seen from a bird’s eye view. However, there are several differences between the 2D small-scale and 3D large-scale environments used in Experiment 1, each potentially impacting the task demands. They include stimulus distance, movement speed, and the characteristics of the visual input during foraging (e.g., limited visibility of individual items at the onset of trial and change of perspective during navigation in a 3D large-scale virtual environment, which may not be present to a similar extent in a 2D small-scale display).

Distance and movement time are two important currencies, often intrinsically associated with one another, that foragers can use to regulate their behaviour. Indeed, they are often used, within the behavioural ecology literature, as proxies for energy consumption that foragers should compare with the energy intake derived from resources gained during foraging [38]. Thus, within the perspective of this study, they would be good candidates for the currencies used by participants in the evaluation of the trade-off between the cognitive and non-cognitive costs associated with their search strategies. For the reasons above, Experiment 2 aimed to determine the possible contribution of movement distance (manipulated as the distance between stimuli) and movement time (manipulated as movement speed) to changes in switching behaviour.

We wanted to avoid possible confounding effects of other variables covarying with the use of 2D versus 3D, such as the extent of changing viewpoint during foraging. Thus, we manipulated stimulus distance and movement speed exclusively within a 2D small-scale display, where participants have a bird’s eye view of the search space.

Moreover, to simplify the experimental design, and enable a more straightforward interpretation of the results, Experiment 2 was divided into two related experiments (2a and 2b). In Experiment 2a we manipulated the distance between the visual stimuli in the display, and in Experiment 2b we manipulated the speed at which the cursor could move between targets. Thus, we assessed the effect of these two variables on the frequency of switches independently. As Experiment 1 confirmed the important role played by stimulus complexity in determining changes in search strategies, stimulus type (feature versus conjunction) was manipulated in both Experiment 2a and Experiment 2b. As Experiment 2 was ran during the COVID-19 pandemic, testing was carried out online using the Gorilla Experiment Builder platform [39,40].

## Experiment 2a

### Method

#### Participants.

Eighty undergraduate students from the University of Leicester were originally recruited to take part online, in exchange for course credits. However, twelve of these participants failed to complete the experiment and were omitted, leaving the dataset with a sample size of sixty-eight individuals (10 males and 58 females). The age range was 18–40 years (*M* = 19.61 years, *SD* = 2.70). All participants reported to have either normal or corrected-to-normal vision. Fifty-nine participants were right-handed and nine were left-handed.

#### Design.

**Fig 4:**
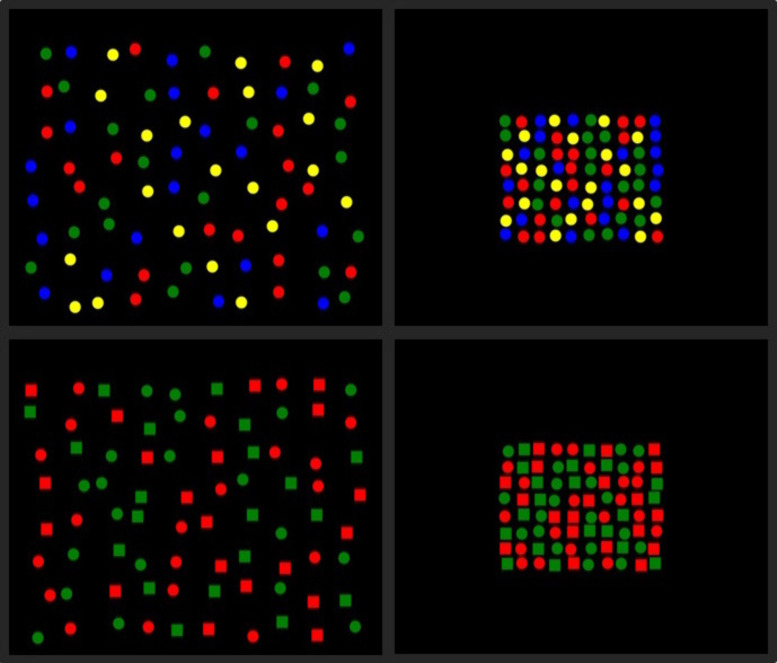
Examples of displays used for each condition of Experiment 2a. Displays of the four conditions used in Experiment 2a: sparse feature (top left), dense feature (top right), sparse conjunction (bottom left), dense conjunction (bottom right). For feature conditions, targets were either red and green circles or blue and yellow circles. For conjunction conditions targets were either red squares and green circles or green squares and red circles. The stimuli which were not targets were always distractors.

This experiment was similar to the 2D small-scale visual foraging condition used in Experiment 1, with the exception of the manipulation of the distance between stimuli. A repeated measures design 2 (Stimulus Distance: dense, sparse) x 2 (Stimulus Type: feature, conjunction) was used (see Fig 4). The sparse foraging conditions presented stimuli which were at double the distance from one another than the stimuli in the dense condition. The feature conditions presented red and green circles as targets among blue and yellow circle distractors. The conjunction conditions presented red squares and green circles as targets with red circles and green squares as distractors. The order of presentation of the four conditions was counterbalanced. Target switching frequency was the dependent variable, whereby the number of switches between target types were analysed.

#### Materials.

The online experiment was developed using Gorilla Experiment Builder [39,40] software, with its web platform (Gorilla.sc). Participants were required to use a personal computer. Evidence suggests that despite attrition issues, online experiments can be comparable in terms of quality of data produced [41–43].

The array of images was fitted to the ‘designated screen area’ as a regular grid, then images were jittered. The designated screen area was the 4:3 area defined by Gorilla (the largest 4:3 area that would fit on the screen) with a border added to keep images away from the edges of the screen. The border was 10% of the width and height of Gorilla’s 4:3 area. Image diameter was fixed at 5% of the designated screen area. When compression was required (0.5 for dense and 1.0 for sparse stimuli arrays), the area that was occupied by the grid was decreased by the specified percentage of the designated screen area. Jittering involved the random displacement of images from their grid positions in the x and y directions. Images were prevented from touching or overlapping by setting the maximum jitter distance to 90% of half the distance between adjacent images in the grid. After the positions of images in the array had been determined, the actual images (each with a colour and shape) were assigned to these positions according to a pseudorandomised sequence. The RGB values were the same as in Experiment 1.

#### Procedure.

The four conditions were counterbalanced by having eight potential order sequences, for conditions which used either red/green targets or blue/yellow targets (hence 16 potential condition sequences) to reduce any possible effect of order and colour as spurious variables. There were 20 trials in each of the four conditions. Participants selected the stimuli by clicking with a mouse.

The procedure was similar to the 2D small-scale foraging condition of Experiment 1, with the two differences being the addition of distance as a variable and testing online via computer and mouse rather than face-to-face with a computer and touchscreen. After giving informed consent to participate, participants were instructed to select 40 targets while avoiding the 40 distractors for each trial. Selections made on the dark space outside of stimuli elicited no effect. Selecting distractor stimuli would immediately end the trial. Each trial was successfully completed when all targets had been selected. The experiment ended when the trials for all four foraging conditions had been either ended because a distractor was selected or successfully completed.

### Results

**Fig 5:**
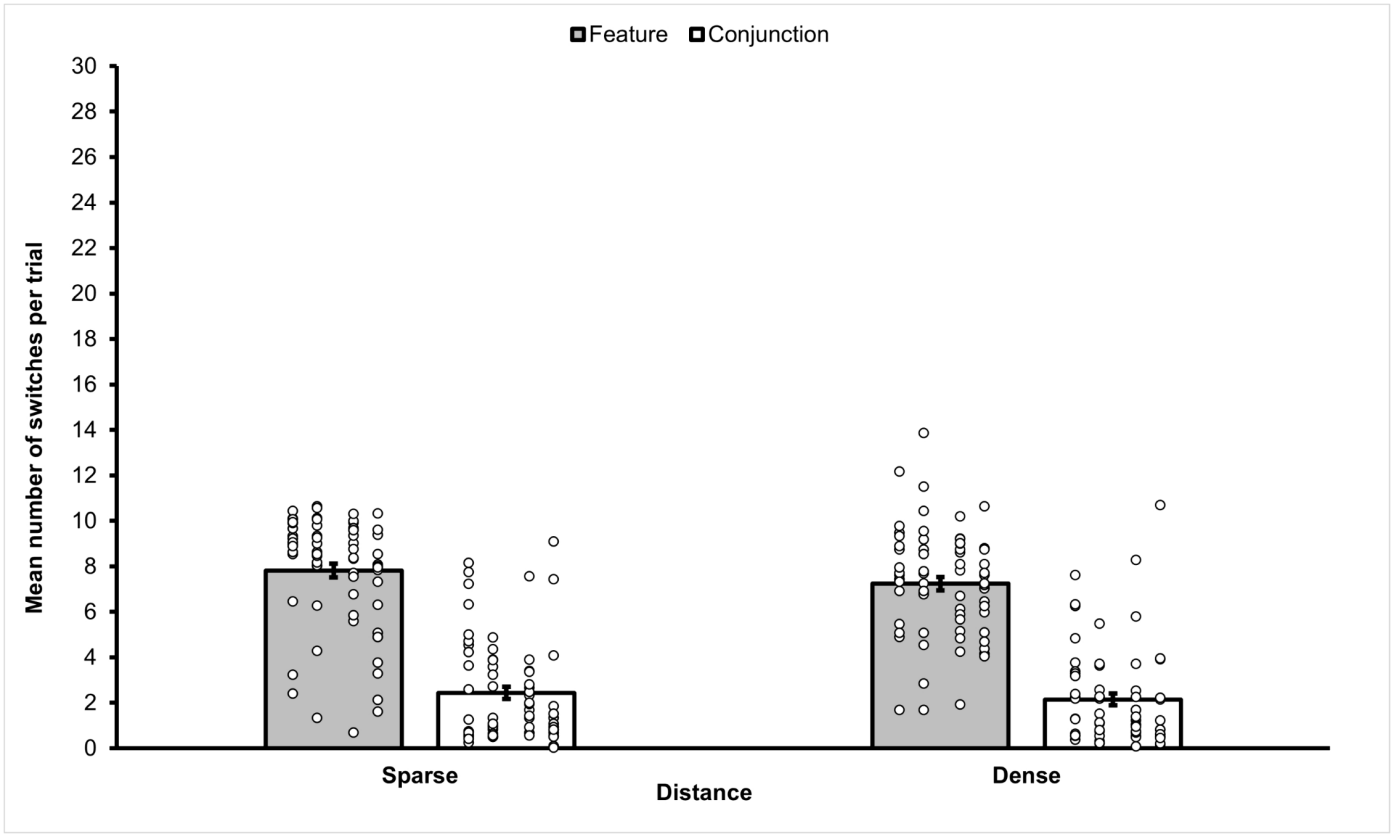
Mean frequency of target switches observed in the different sub-conditions of Experiment 2a. Mean frequency of target switches for each sub-condition of Experiment 2a. From the figure is can be observed that the sparse conditions statistically significantly elicited more switches than dense conditions. Additionally, feature conditions statistically significantly elicited more switches than conjunction conditions. Error bars represent standard error of the mean. Small circles represent individual scores.

Following a procedure adopted by other authors [18], the dataset was cleaned up by omitting data from all trials which ended because a distractor was selected. We removed 1057 trials (19.43% of trials), leaving 4383 trials for analysis. In more detail, we removed 153 trials (11.25%) in the Sparse Feature condition, 284 trials (20.88%), in the Sparse Conjunction condition, 242 trials (17.65%) in the Dense Feature condition, and 378 trials (27.79%) in the Dense Conjunction condition. The mean frequency of target switches observed in each of the four conditions of Experiment 2a, is presented in Fig 5.

A 2 (Distance: sparse, dense) x 2 (Stimulus Type: feature, conjunction) repeated measures ANOVA on the frequency of target switching was conducted. It revealed a significant main effect of Distance (*F*(1,67) = 6.302, *p* = .017, *η*_*p*_^2^ = .08), with higher mean switches in the sparse condition (*M* = 5.13, *SEM* = .24, 95% *CI* [4.65, 5.60]) compared to dense condition (*M* = 4.69, *SEM* = .21, 95%, *CI* [4.28, 5.10]). However, it can be noted that effect size was small. A highly significant main effect of Stimulus Type was also observed (*F*(1,67) = 288.092, *p* < .001, *η*_*p*_^2^ = .81), with a higher mean frequency of switches in the feature condition (*M* = 7.53, *SEM* = .26, 95% *CI* [7.00, 8.05]) compared to the conjunction condition (*M* = 2.29, *SEM* = .25, 95% *CI* [1.79, 2.79]). The interaction between Distance and Stimulus Type was not statistically significant (*F*(1,67) =.777, *p* = .374, *η*_*p*_^2^ = .01).

A 2 (Distance: sparse, dense) x 2 (Stimulus Type: feature, conjunction) repeated measures ANOVA on the number of errors performed in the different conditions revealed that Dense conditions resulted in significantly more errors (*M* = 4.544, *SEM* = .467, 95% *CI* [3.474, 5.614]) than Sparse conditions (*M* = 3.213, *SEM* = .458, 95% *CI* [2.163 4.263]), (*F*(1,67) = 13.499, *p* < .001, *η*_*p*_*²* = .168). Conjunction stimuli produced significantly more errors (*M* = 4.868, *SEM* = .527, 95% *CI* [3.658, 6.077]) than Feature stimuli (*M* = 2.890, *SEM* = .415, 95% *CI* [1.939, 3.840]), (*F*(1, 67), = 22.191, *p* < .001, *η*_*p*_*²* = .249). The interaction between Density and Stimulus Type was not statistically significant (*F*(1, 67), = .018, *p* = .893, *η*_*p*_*²* = .001). Pearson’s R correlations between the frequency of switching and path length in completed trials did not prove statistically significant for the Dense-feature condition (*r*(66) = −.062, *p* = .617), Dense-conjunction condition (*r*(66) = −.162, *p* = .188), Sparse-conjunction condition (*r*(66) = −.223, *p* = .068) and Sparse-feature condition (*r*(66) =.113, *p* = .359).

### Discussion

In Experiment 1, it was observed that foraging in 3D immersive large-scale environments induced participants to switch more than when foraging in a 2D small-scale environment seen from a bird’s eye view. In addition to the scale of environment and associated relative movement distance, the 3D and 2D environments in Experiment 1 caused variations in other aspects of foraging, such as the continuous change of viewpoint entailed by moving in the immersive 3D environment. Thus, the main aim of Experiment 2a was to determine whether increasing the distance between stimuli, and therefore the length of the movements required to travel from one to another, would by itself induce participants to increase their switching frequency.

The results showed that this is the case since foraging among the sparsely arranged stimuli produced a higher frequency of switching compared to foraging among densely arranged stimuli, when the dimensionality of the foraging environment was kept constant and in 2D.

Moreover, the results of Experiment 2a confirmed that foraging among stimuli defined by a conjunction of visual properties (colour and shape) induces fewer switches between targets than stimuli defined by only a single property (colour). This is likely due to increased load or cognitive costs derived from maintaining more complex templates in working memory, as suggested also by Kristjánsson et al. [9,18]. Conjunction conditions proved more difficult since more errors were observed there. Dense conditions also produced more errors than sparse conditions and a contribution of an effect of crowding (targets becoming more difficult to identify when surrounded by other stimuli nearby) to this difference cannot be ruled out. Thus, the interpretation of the results of this experiment is less straightforward than that of Experiment 1, given the possible contribution of difficulty and crowding to the increase of switch frequency observed in the present experiment.

Experiment 2b investigated whether or not movement speed in itself affects target switching behaviour. As stimulus distance were kept constant in the different conditions of 2b crowding and discriminability could not account for any differences in target switching observed there.

## Experiment 2b

### Method

#### Participants.

Eighty undergraduate students from University of Leicester were initially recruited to take part. However, 20 of these participants did not complete the experiment and had to be excluded, leaving the dataset with a sample size of sixty individuals (14 males and 46 females). The age range was 18–26 years (*M* = 19.36 years, *SD* = 1.38). All participants reported either normal or corrected-to-normal vision. Fifty-three participants were right-handed and seven were left-handed. They received course credits for taking part in the study.

#### Design.

**Fig 6:**
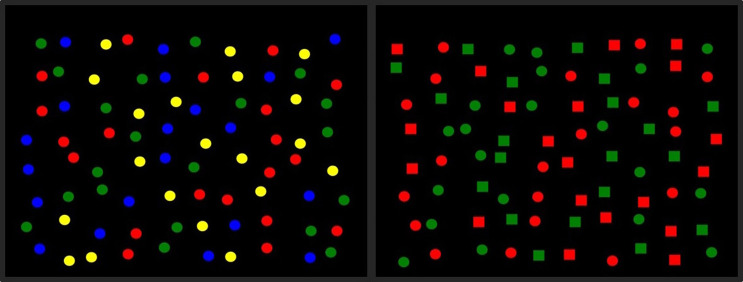
Examples of displays used in each condition of Experiment 2b. The targets for the feature (left) and conjunction (right) conditions were the same as in Experiment 1.

The design was similar to Experiment 2a; however, in Experiment 2b the independent variables were (1) Movement Speed, dichotomised into fast and slow cursor speed, and (2) Stimulus Type (feature: red and green circle targets with blue and yellow circle distractors; conjunction: red square and green circle targets with red circle and green square distractors). Examples of displays for these conditions are presented in Fig 6. The design was a 2 (Movement Speed: fast, slow) x 2 (Stimulus Type: feature, conjunction) repeated measures design, so that four experimental conditions were used: 1) fast feature, 2) slow feature, 3) fast conjunction, and 4) slow conjunction. The order of presentation of the four conditions was counterbalanced. Half of the participants received red/green targets and half blue/yellow targets. The frequency of target switching was analysed to assess differences in foraging strategy between the conditions.

#### Materials.

The materials used were identical to those used in Experiment 2a. The only difference was the manipulation of the speed at which the cursor could move. We used two speeds: slow and fast. The fast movement speed was the same as the speed of movement used in the 2D environment of Experiment 1. The slow movement speed was half the speed of the fast movement. As set, the minimum time that it could take to move the cursor between stimuli in the slow condition was 1.42 seconds. By contrast in the fast condition, it was 0.71 seconds.

#### Procedure.

The procedure was similar to that of Experiment 2a, with the exception that movement speed was manipulated instead of distance. As in Experiment 2a, participants were instructed to select 40 targets while avoiding the 40 distractors for each trial. There were 20 trials in each of the four conditions. Participants were informed that cursor speed would change in some trials and instructed that they should not change the cursor speed on their PC.

### Results

**Fig 7:**
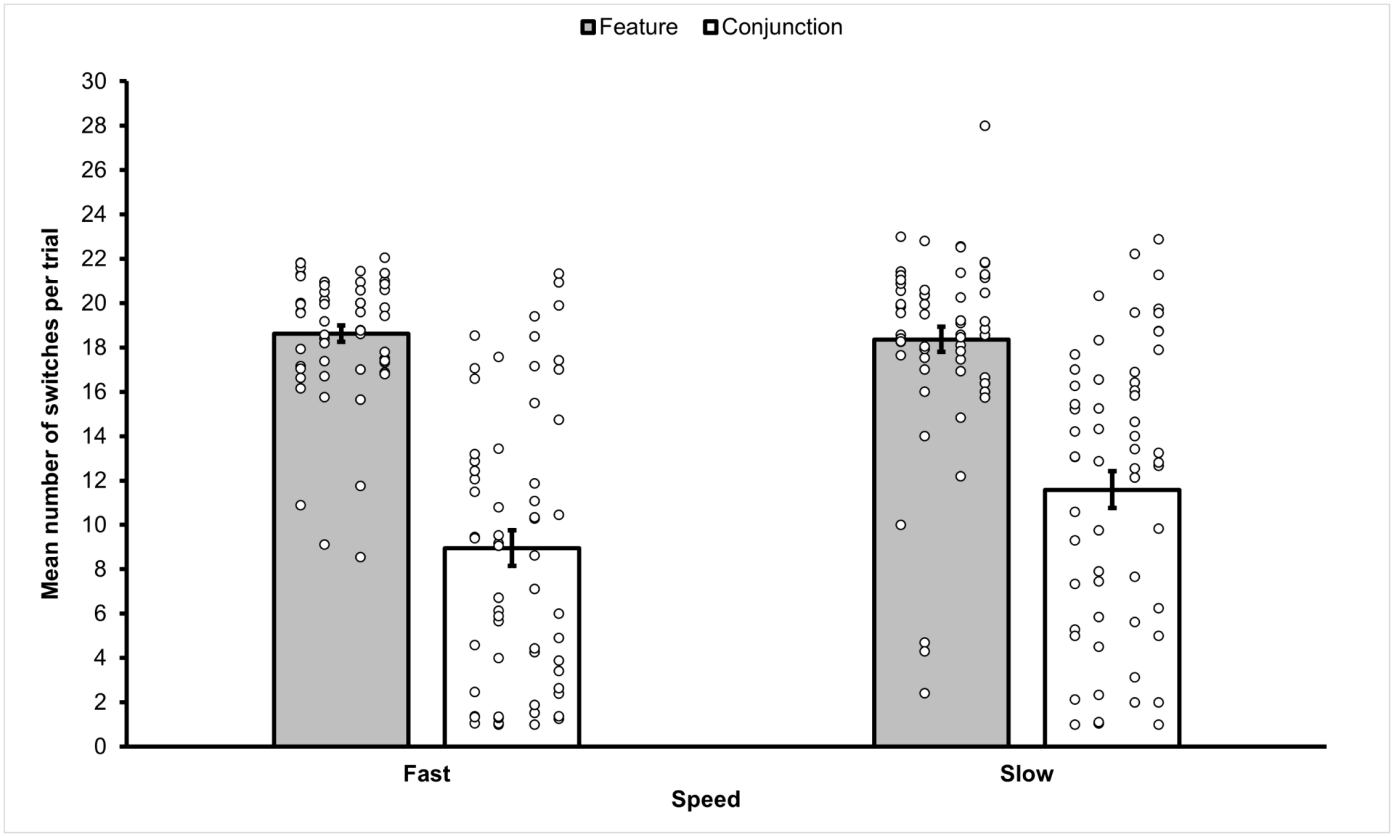
Mean frequency of switches between target categories observed in the different sub-conditions of Experiment 2b. Target switches observed in the different sub-conditions of Experiment 2b. Overall feature conditions elicited statistically significantly more switches than conjunction conditions. The figure also shows that the effect of movement speed is evident in the conjunction conditions, with statistically significantly more switches observed in the slow conjunction condition compared to the fast conjunction condition. Error bars represent one standard error of the mean. Small circles represent individual scores.

Before the analyses, the data was cleaned up by removing trials that terminated before completion because a distractor was selected. A total of 1001 trials (20.85%) were removed, leaving 3799 trials for analysis. In more detail, for the Fast Feature condition we removed 128 trials (10.67%), 315 trials (26.25%) in the Fast Conjunction condition, 202 trials (16.83%) in the Slow Feature condition, and 356 trials (29.67%) in the Slow Conjunction condition. The mean frequency of switches observed in each of the four conditions (fast feature, slow feature, fast conjunction, slow conjunction) of Experiment 2b is reported in Fig 7.

A 2 (Movement Speed: fast, slow) x 2 (Stimulus Type: feature, conjunction) repeated measures ANOVA on the frequency of switching was carried out. The ANOVA revealed a significant effect of Movement Speed (*F*(1,59) = 8.206, *p* = .006, *η*_*p*_^2^ = .12), with a higher mean frequency of switches in the slow condition (*M* = 14.97, *SEM* = .61, 95% *CI* [13.76, 16.19]) compared to the fast condition (*M* = 13.79, *SEM* = .488, 95% *CI* [12.81, 14.76]). A significant main effect of Stimulus Type was also observed (*F*(1,59) = 162.793, *p* < .001, *η*_*p*_^2^ = .73), with a higher mean frequency of switches in the feature (*M* = 18.49, *SEM* = .40, 95% *CI* [17.70–19.28]) compared to the conjunction condition (*M* = 10.27, *SEM* = .758, 95% *CI* [8.75–11.79]). There was also a statistically significant interaction between Movement Speed and Stimulus Type (*F*(1,59) = 12.799, *p* = .001, *η*_*p*_^2^ = .18). Paired sample t-tests with Bonferroni correction showed that the interaction could be explained by the presence of a significant difference between the number of switches performed in the fast (*M* = 8.95, *SEM* = .81) and slow (*M* = 11.59, *SEM* = .83) conjunction conditions (*t*(59) = −4.25, *p* < .001), in absence of a difference be*t*ween the fast (*M* = 18.62, *SEM* = .37) and slow (*M* = 18.36, *SEM* = .56) feature conditions (*t*(59) =.48, *p* = .631).

A 2 (Movement Speed: fast, slow) x 2 (Stimulus Type: feature, conjunction) repeated measures ANOVA revealed that significantly more errors were performed in the Slow conditions (*M* = 6.786, *SEM* = .820, 95% *CI* [4.907, 8.664]) than Fast conditions (*M* = 5.25, *SEM* = .639, 95% *CI* [3.787, 6.713]), (*F*(1,59) = 14.052, *p* < .001, *η*_*p*_*²* = .169). Conjunction foraging produced significantly more errors (*M* = 7.436, *SEM* = 0.748, 95% *CI* [5.722, 9.149]) than Feature foraging (*M* = 4.6, *SEM* = .707, 95% *CI* [2.979, 6.221]), (*F*(1, 59), = 63.581, *p* < .001 *η*_*p*_*²* = .480). The interaction between speed and foraging type was not statistically significant (*F*(1, 59), = 1.967, *p* = .165, *η*_*p*_*²* = .028).

Correlation analyses revealed statistically significant negative relationships of mean number of switches and mean path length found in the fast-conjunction condition (*r*(58) = −.417, *p* < .001), slow-feature condition (*r*(58) = −.369, *p* = .004) and slow-conjunction condition (*r*(58) = −.374, *p* = .003). However, there was no statistically significant relationship between switches and path length in fast-feature condition (*r*(58) = −.072, *p* = .584).

### Discussion

Experiment 2b confirmed that participants are likely to switch more in feature conditions, presumably due to the relatively low cognitive costs of simultaneously holding multiple simple templates in working memory to guide search. However, in the conjunction condition, where templates are complex and impose high cognitive costs in terms of working memory, participants deploy search strategies characterised by fewer switches. These latter strategies do not require holding several templates in mind at the same time for prolonged periods. When foraging for targets defined by a conjunction of features, participants spontaneously increased their frequency of switching only when the cursor speed was slow. Thus, moving at slow speed seems to provide an incentive to trade cognitive costs for the opportunity to select nearby targets of a different category and, by doing so, counteract the considerable increase in overall search time that would be entailed by travelling longer distances (since some targets would not be selected as soon as they are encountered and would require returning to their location later) at low speed. The notion that switching more often results in a shorter search path was confirmed for most conditions, with the exception of the fast feature condition. Importantly, in this experiment we observed more switches in conditions where participants made more errors. If errors provide an indication of the difficulty of the conditions, then added difficulty cannot explain the decrease in the frequency of target switching. Similarly, as the distance between the stimuli was kept constant across conditions any effects of crowding and discriminability on target switching can be ruled out.

Experiments 2a and 2b were both carried out in 2D small-scale foraging conditions not dissimilar to the 2D conditions of Experiment 1, and where participants had a bird’s eye view of the search space throughout the task. Experiment 3 investigated the extent to which changes of distance and movement speed are enough to cause changes in foraging behaviour in 3D environments as well.

## Experiment 3

In Experiment 2 we manipulated movement speed and distance to determine if these variables had an effect when the foraging environment was kept constant in 2D. The rationale for the experiment was to disentangle the effects of distance travelled and movement speed from other factors that may have contributed to the differences in search strategies observed in Experiment 1, where foraging in small-scale 2D and large-scale 3D environments was compared. An example of a confounding factor would be the continuous change of viewpoint entailed by foraging within an immersive 3D navigational environment but absent in instances of visual foraging in 2D, where the forager maintains a fixed bird’s eye view of the search space.

We decided to start with experiments featuring the 2D small-scale display due to restrictions on face-to-face testing imposed by the COVID-19 pandemic at the time of testing. Testing remotely using the Gorilla platform (Gorilla.sc) [39,40] in Experiment 2 allowed us to implement the task successfully and highlighted some important effects of distance and speed of travel even in a small display, seen from a bird’s eye view. Nonetheless, it remained to be determined whether or not the observed effects are confined to a 2D small-scale display. Thus, Experiment 3 evaluated whether variations of distance and speed affected participants’ search strategies within a large-scale 3D foraging environment.

Since in Experiment 2 we observed different patterns of results for foraging for targets identifiable on the basis of single feature and for targets which required a conjunction of features to be identified, we decided to use each of these two types of foraging in separate experiments. Therefore, Experiment 3a focused on the effects of distance and movement speed in displays of stimuli defined by a single property (colour), whereas Experiment 3b focused on stimuli defined by a conjunction of two properties (colour and shape).

### General Methods

Given the similarity of Experiments 3a and 3b, we describe the general methodology shared by both experiments here. More specific aspects of the methodology, relevant to each of the two experiments, are described below in the methods section of Experiment 3a and 3b, respectively.

#### Design.

Both Experiment 3a and 3b used a 2 (Distance: sparse, dense) x 2 (Movement Speed: fast, slow), repeated measures design, with all conditions presented in the 3D large-scale environment.

#### Materials.

The materials were the same as those used in the 3D large-scale foraging task of Experiment 1. Vizard 5 [36] was used to create the virtual environment, which was displayed on an ASUS VG278HE PC monitor (60 cm wide x 34 cm high), via 3D glasses (NVIDIA 3D Vision 2). A PlayStation 4 controller was used for navigation.

**Fig 8:**
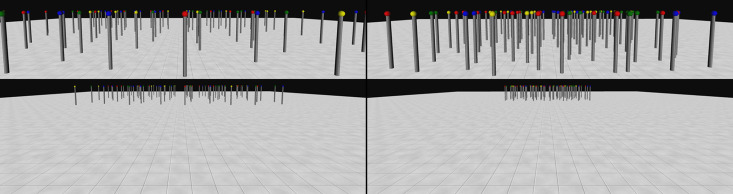
Examples of 3D large-scale sparse feature condition and dense feature condition used in Experiment 3a. Example of the environment used for the sparse feature condition (bottom left), with a zoomed-in image (top left). Example of the dense feature condition (bottom right) with a zoomed-in image (top right). Zoomed in images of each condition are presented here to allow a better visualisation of the stimuli. They were not displayed as part of the experimental manipulations. However, similar viewpoints would have been experienced by the participants when at different distances from the array while moving in the environment. The size of the environment used in sparse and dense conditions was the same. Only the spacing of the stimuli arrangement differed.

In both Experiments 3a and 3b, an array of 80 poles was displayed in the centre of a virtual room. Figs 8 and 10 show examples of how the search space would be seen from the participant at the start of a trial of each condition. Please note, however, that the figure had to be slightly distorted to enable us to present it as a 2D figure in this article. Sparse conditions were identical to the 3D large-scale conditions of Experiment 1, whereas dense conditions featured a higher density arrangement of the stimuli.

**Fig 9:**
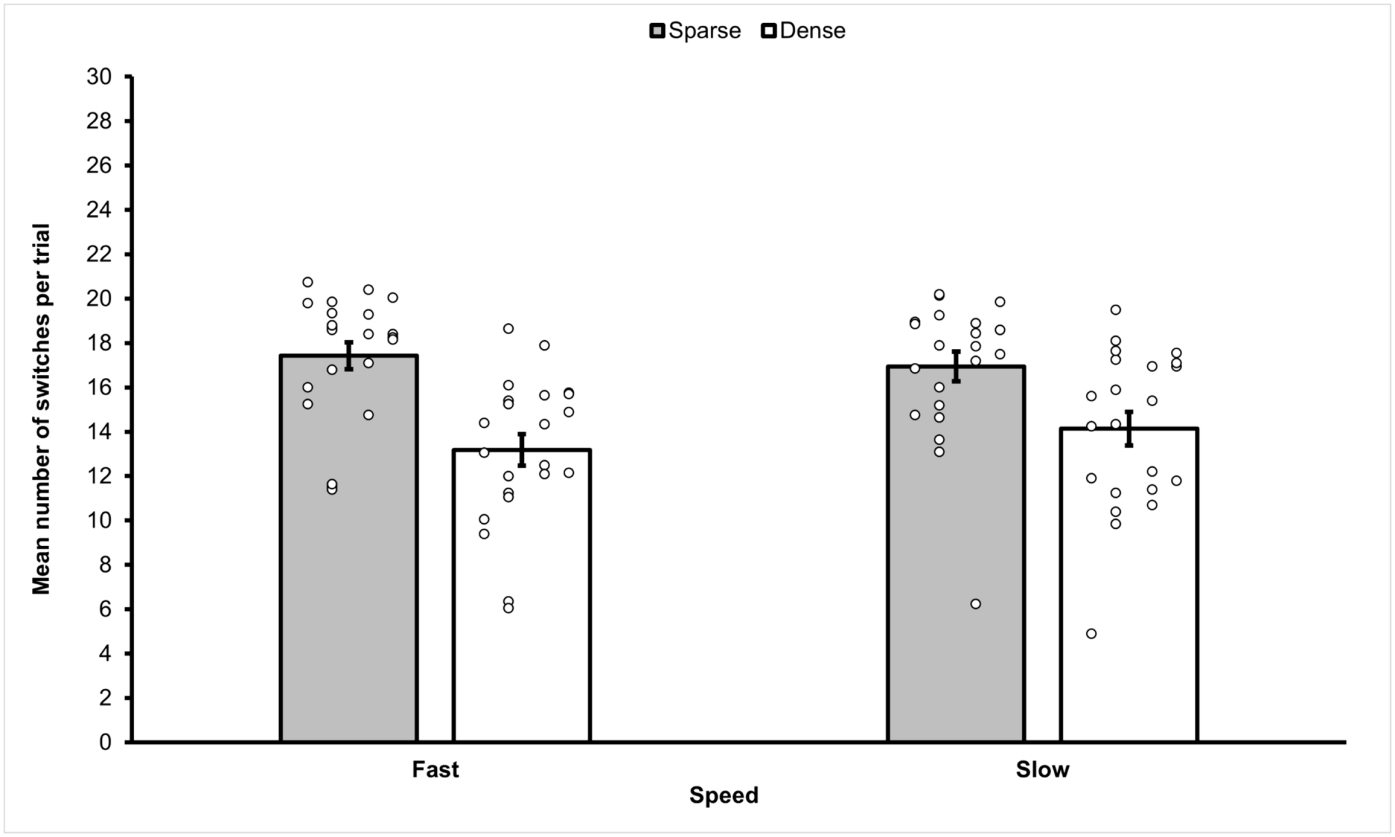
Mean frequency of target switches observed in the different sub-conditions of Experiment 3a. Mean number of switches observed following the manipulation of Stimulus Distance (sparse and dense) and Movement Speed (fast and slow) in Experiment 3a. The figure shows sparse conditions eliciting statistically significantly more switches than dense conditions. The differences between fast and slow speed conditions were not statistically significant (see text for details). Error bars represent standard error of the mean. Small circles represent individual scores.

The visual display was identical in the fast and slow conditions. The participant could see the whole array of poles from a starting position close to one wall of the room. Room size was kept the same for the dense and sparse arrays (approximately 49.2 x 49.2 vm across, and 7.3 vm high). Because the room size was fixed, the starting distance from the array would be greater for the dense array than for the sparse array as a result. The distance between poles was 2 vm in the sparse condition and 1 vm in the dense condition. The maximum distance at which a pole was selectable needed to be less than half the distance between poles, to avoid that more than one pole could be selected at the same time. This distance was required to be the same for the dense and sparse arrays, so it was fixed at the size required to satisfy the above condition for the dense array (0.45 vm). Jittering involved the random displacement of poles from their grid positions in the x and y directions. Poles were prevented from touching or overlapping with each other by setting the maximum jitter distance to 80% of half the distance between adjacent poles in the grid (0.736 vm for the sparse array and 0.336 for the dense array). After the positions of the poles in the array had been determined, the actual poles (with a colour and shape) were assigned to these positions according to a randomised sequence. The RGB values were the same as in Experiments 1, 2a and 2b.

### Procedure

The procedure was similar to that used for Experiment 1. Participants were required to navigate a 3D large-scale environment and select target stimuli using a PlayStation 4 controller. As with Experiment 1, selecting a stimulus required the participant to move towards a pole within the virtual space. Once they reached the proximity of 0.45 vm of a pole, a prompt “select pole?” appeared above the pole. Participants could then decide to select the pole by pressing a button (R1) on the controller. Selections made outside the range of 0.45 vm from any pole (whereby no selection prompt would be visible) had no effect. Each trial ended when all targets (40) had been selected, unless the participant selected a distractor. Selecting a distractor caused the trial to end immediately.

Distance was manipulated by changing the density of the stimuli. In the sparse array the stimuli were at twice the distance from each other than in the dense array. This ratio was the same as that used for dense and sparse arrays in Experiment 2a. Examples of the arrays used in the two conditions are presented in Fig 8 (for Experiment 3a) and 10 (for Experiment 3b).

Speed was manipulated as follows. Maximum speed of movement in the fast condition was set to 4 vm/s (virtual metres per second), which was the same maximum speed used in Experiment 1. In the slow conditions maximum speed was 2 vm/s (50% slower than in the fast condition). Videos showing examples of trials for the different conditions of Experiment 3 are available online from the Open Science Framework https://osf.io/pc5gt/.

## Experiment 3a

### Method

#### Participants.

Twenty-two undergraduate students (four males and 18 females) from the University of Leicester participated in the experiment in exchange for course credits. The age range was 18–40 years (*M* = 20.45 years, *SD* = 5.27). All participants had either normal or corrected-to-normal vision. Eighteen participants were right-handed and four were left-handed. None of them had taken part in previous experiments.

#### Procedure.

The procedure was as described in the general methods above. The stimuli were as those described for the feature conditions of Experiment 1, with targets being red and green spheres or blue and yellow spheres. Examples of the different conditions used for this experiment are presented in Fig 8.

### Results

Before the analyses, the dataset was cleaned up by removing trials which ended because a distractor was selected. A total of 325 trials (18.47%) were removed, leaving 1435 trials for analysis. In more detail, the number of trials removed for each condition was: 35 (7.95%) for the Sparse Fast condition; 46 trials (10.45%) for the Sparse Slow condition; 132 trials (30%) for the Dense Fast condition, and 112 (25.45%) for the Dense Slow condition.

The mean number of switches observed in the different conditions of Experiment 3a are reported in Fig 9.

A 2 (Distance: sparse, dense) x 2 (Movement Speed: fast, slow) repeated measures ANOVA on the frequency of switching revealed a statistically significant main effect of Distance (*F*(1,21) = 49.837, *p* < .001, *η*_*p*_^2^ = .70), with a higher mean frequency of switches observed in sparse conditions (*M* = 17.18, *SEM* = .50, 95% *CI* [16.14–18.23]) compared to the dense conditions (*M* = 13.66, *SEM* = .62, 95% *CI* [12.36, 14.95]). By contrast, the main effect of Movement Speed was not statistically significant (*F*(1,21) =.150, *p* = .702, *η*_*p*_^2^ = .07) as the means for the slow (*M* = 15.54, *SEM* = .541, 95% *CI* [14.21, 16.86]) and the fast (*M* = 15.30, *SEM* = .637, 95% *CI* [14.18, 16.43]) conditions did not differ. The interaction between Distance and Movement Speed was also not significant (*F*(1,21) = 1.978, *p* = .174, *η*_*p*_^2^ = .09).

A 2 (Distance: sparse, dense) x 2 (Movement Speed: fast, slow) repeated measures ANOVA revealed that dense conditions had more error trials (*M* = 5.55, *SEM* = .65, 95% *CI* [3.98, 4.98]) than Sparse conditions (*M* = 1.84, *SEM* = .617, 95% *CI* [0.711, 2.971]), (*F*(1,21) = 35.957, *p* < .001, *η*_*p*_*²* = .631). There was no statistically significant difference between Fast (*M* = 3.795, *SEM* = 0.492, 95% *CI* [2.608, 4.983]) and Slow conditions (*M* = 3.591, *SEM* = 0.617, 95% *CI* [2.102, 5.080]), (*F*(1, 21), = .119, *p* = .733, *η*_*p*_*²* = .006). The interaction between density and speed was not statistically significant (*F*(1, 21), = .2098, *p* = .162, *η*_*p*_*²* = .091).

There was a statistically significant negative correlation between number of switches and path length in sparse-fast conditions (*r*(20) = −.754, *p* < .001), sparse-slow conditions (*r*(20) = −.522, *p* = .013), and dense-slow conditions (*r*(20) = −.445, *p* = .038). However, the relationship between switches and path length was not statistically significant in dense-fast conditions (*r*(20) = −.417, *p* = .054).

### Discussion

The findings of Experiment 3a revealed that distance significantly affected target switching. This is congruent with the findings of Experiment 2a and shows that, also within a 3D environment, increasing the distance between targets encourages people to engage in more cognitively costly strategies. The fact that in most conditions there was a significant negative correlation between frequency of switches and distance travelled to complete a trial suggest that people increased the frequency of switching, to reduce movement distance. Nonetheless, in this experiment we also observed more errors, resulting in the termination of the trials in dense conditions. This could be an indication of a contributing effect of crowding there. However, the significant effect of movement speed observed in Experiment 2b did not emerge in Experiment 3a. Thus, within the context of this experiment, movement distance seems to have a greater influence than speed in determining changes in foraging strategies as assessed by comparing the frequency of spontaneous switching between stimulus categories. This could imply that with reference to the extra cognitive costs of foraging, distance is perceived as the most important currency to use to offset the cognitive load imposed by foraging strategies characterised by a higher frequency of switches between target categories. As this experiment used stimuli defined by a single feature, the overall frequency of switches was relatively high. Experiment 2b showed that the differences between conditions were more prominent and statistically significant when participants foraged for targets defined by a conjunction of features. Whether or not foraging for targets defined by a conjunction of features resulted in a more prominent difference between conditions, as well as a potentially different result for the impact of speed on foraging strategy, was explored in Experiment 3b.

## Experiment 3b

### Method

#### Participants.

Twenty undergraduate students (two males and 18 females) from University of Leicester participated in the experiment in exchange for course credits. The age range was 18–33 years (*M* = 19.30 years, *SD* = 3.28). All participants had either normal or corrected-to-normal vision. Eighteen participants were right-handed and two were left-handed.

#### Procedure.

**Fig 10:**
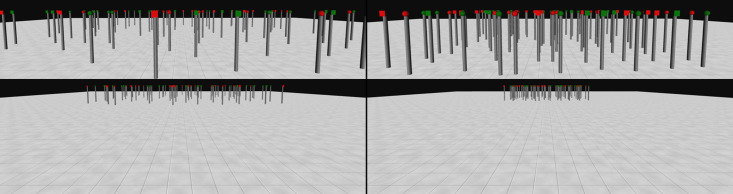
Examples of 3D large-scale sparse conjunction condition and dense conjunction condition used in Experiment 3b. Example of the environment used for the sparse conjunction condition (bottom left), with a zoomed-in image (top left). Example of the dense conjunction condition (bottom right) with a zoomed-in image (top right). Zoomed-in images of each condition are presented here to allow a better visualisation of the stimuli and were not displayed as part of the procedure. However, similar viewpoints would have been experienced by the participants when at different distances from the array while moving in the environment. The size of the environment used in sparse and dense conditions was the same. Only the spacing of the stimuli arrangement differed.

The procedure was as described in the general methods above and resembled that used for the 3D conjunction condition of Experiment 1. So, the targets shared one of their two properties (colour or shape) with distractors. Apart from the use of stimuli (red cubes, red spheres, green cubes, green spheres), that required a conjunction of features to be identified, the methods used for Experiment 3b were identical to those used for Experiment 3a (see Fig 10).

### Results

Before the analyses, the data were cleaned up by removing trials which ended because a distractor was selected. We removed 295 trials (18.44%), leaving 1305 trials for analysis. In more detail, for the Sparse Fast condition we removed 30 trials (7.50%), 48 trials (12%) in the Sparse Slow condition, 116 trials (29%) in the Dense Fast condition, and 101 trials (25.25%) in the Dense Slow condition.

**Fig 11:**
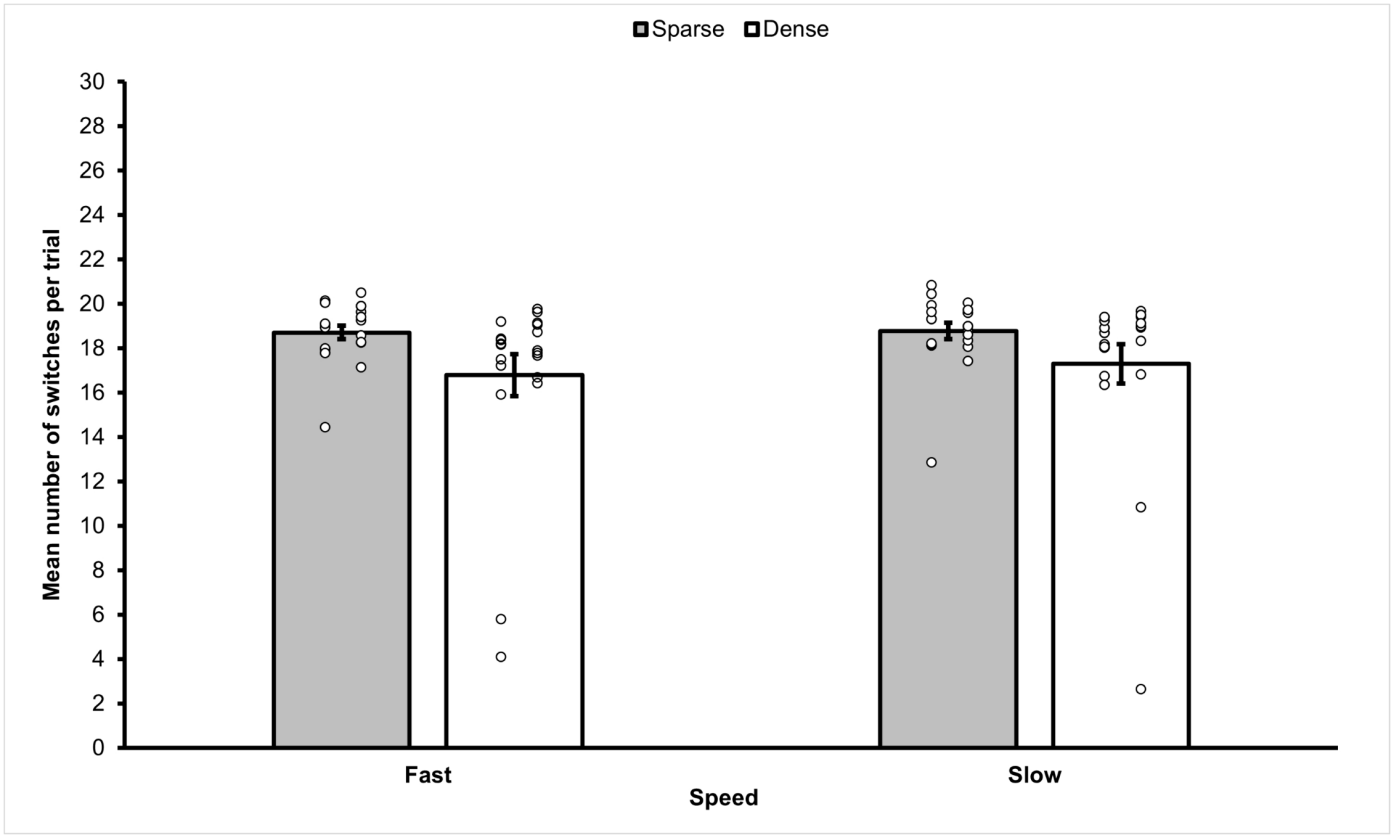
Mean frequency of target switches observed in the different sub-conditions of Experiment 3b. From the figure it can be observed that the sparse conditions elicited a higher frequency of switches than dense conditions. Differences between the mean number of switches in the fast and slow conditions were smaller and did not prove statistically significant (see text for details). Error bars show standard error of the mean. Small circles represent individual scores.

The mean frequency of target switches for the different conditions of Experiment 3b are reported in Fig 11.

A 2 (Distance: sparse, dense) x 2 (Movement Speed: fast, slow) repeated measures ANOVA on the frequency of switching was carried out. Similarly to what was observed in Experiment 3a, the ANOVA revealed a statistically significant main effect of Distance (*F*(1,19) = 4.595, *p* = .045, *η*_*p*_^2^ = .20), with a higher mean frequency of switches observed in the sparse condition (*M* = 18.74, *SEM* = .26, 95% *CI* [18.202, 19.28]) compared to the dense condition (*M* = 17.04, *SEM =* .88, 95% *CI* [15.20, 18.88]). As for Experiment 3a, there was no effect of Movement Speed (*F*(1,19) = 1.667, *p* = .212, *η*_*p*_^2^ = .08), since the difference between the mean number of switches recorded in the slow (*M* = 18.04, *SEM* = .50, 95% *CI* [17.00, 19.07]) and fast (*M* = 17.75, *SEM* = .56, 95% *CI* [16.58, 18.91]) condition did not prove statistically significant. The interaction between Distance and Movement Speed was also not statistically significant (*F*(1,19) =.317, *p* = .580, *η*_*p*_^2^ = .02).

A 2 (Distance: sparse, dense) x 2 (Movement Speed: fast, slow) repeated measures ANOVA revealed that dense conditions had more error trials (*M* = 5.425, *SEM* = .535, 95% *CI* [4.124, 6.726]) than Sparse conditions (*M* = 1.900, *SEM* = .320, 95% *CI* [1.122, 2.678}), (*F*(1, 19) = 52.912, *p* < .001, *η*_*p*_*²* = .736). There was a statistically significant difference between Fast (*M* = 3.650, *SEM* = .492, 95% *CI* [2.452, 4.848]) and Slow conditions (*M* = 3.675, *SEM* = .383, 95% *CI* [2.744, 4.606]) (*F*(1, 19), = .119, *p* = .003, *η*_*p*_*²* = .000). The interaction between density and speed was not statistically significant (*F*(1, 19), = 4.023, *p* = .059, *η*_*p*_*²* = .175).

There was a statistically significant negative correlation between number of switches and path length in the dense-fast condition (*r*(18) = −.601, *p* = .005). However, the relationship between switches and path length was not statistically significant in the sparse-fast condition (*r*(18) =.05, *p* = .835), the sparse-slow condition (*r*(18) = −.155, *p* = .514) and the dense-slow condition (*r*(18) = −.153, *p* = .521).

### Discussion

The findings of Experiment 3b revealed that distance between items significantly affected target switching. By contrast, even when foraging for targets defined by a conjunction of features, participants did not alter their foraging strategies as a function of movement speed. Thus, the effect of distance seems to be very robust as it emerged in a 3D foraging environment in this experiment, which required foraging for targets defined by a conjunction of features, and in Experiment 3a, which required foraging for targets identifiable by a single feature. A similar effect of distance had also emerged when participants were foraging in a 2D environment in Experiment 2a. However, the frequency of switching was higher in dense conditions, which also registered a higher number of errors that resulted in the termination of the trial. Therefore, a contribution of an effect of crowding or difficulty on the reduced switching cannot be completely ruled out.

The effect of movement speed failed to emerge in our 3D navigational foraging environment. Reducing the maximum speed at which participants could travel did not make them perform more switches. This is likely due to the very high number of switches that participants spontaneously performed in the 3D environment overall. Nonetheless, the fact that distance had an effect in 3D foraging suggests that the lack of an effect of movement speed could not be entirely explained by a ceiling effect. Rather, it seems that the distance between targets provides a stronger incentive for participants to engage in strategies which have a higher cognitive cost. However, sample size for this experiment was slightly smaller than that of the other experiments. Therefore, we cannot exclude the possibility that the effect of speed may have proved significant with a larger sample.

## General Discussion

This study featured a set of five interlinked experiments. They determined the extent to which people spontaneously modulate their foraging strategies on the basis of variations of the characteristics of the environment and other search parameters related to different instances of foraging. We tested the hypothesis that a higher frequency of switches, indicative of the use of cognitively effortful strategies, should be observed when the spatial scale of the foraging environment and its implicit demands, make their use worthwhile.

Our results provide key information on the factors which make people engage in cognitively costly visual foraging strategies requiring frequent switches between memory templates. Following the tenets of several highly influential lines of research [9,18,29,44–46], we used the variations in the frequency of switching between target categories as an indicator of changes in strategy. Kristjánsson et al. [14] demonstrated strategy changes by observing more switches in trials with shorter time limits. According to Kristjánsson et al. [14], in these shorter trials, participants deploy bursts of high-level concentration which enable them to use their full working memory capacity and select more targets per unit of time. Thus, the incentive for using these more effortful strategies would be provided by the increased likelihood of completing an exhaustive search of all the targets in shorter trials. According to Kristjánsson et al. [18], when there is no time limit, there would be no incentive to use effortful strategies because the forager will eventually search the whole display and always select all the targets.

The pattern of switching that we observed across the experiments indicate that shifts towards more cognitively effortful strategies occur even without explicitly imposed time limits on search. This finding provides further support for the notion of a flexible use of working memory capacity in foraging. Our results are consistent with and extend the findings that Kristjánsson et al. [18] obtained by manipulating time limits in 2D small-scale visual displays. The procedure adopted in this study enabled us to overcome some problems associated with the manipulation of search time limits. In fact, the manipulation of time limits can produce confounds between the effects of time limit *per se* and the frequency of switching. When trials are interrupted because a time limit has been reached, trials with longer time limits would contain more switches just because they contain more target selections overall. Moreover, a tendency to switch more often at the trial outset, or later in the trial, could also lead to a different switching frequency for trials with different time limits, even in absence of a strategy change by the participants. The above considerations led Kristjánsson et al. [18] to carry out extensive analyses at different time points to rule out possible confounds and establish that their results truly reflected a change of strategy motivated by the task demands. Our results confirm that people can flexibly alter their strategies as a function of task demands with manipulations that are immune to these problems.

Moreover, our results indicate a trade-off between cognitive and movement costs which proved difficult to dissociate in previous studies focused on tasks demands imposed by different spatial layouts of goal locations [11–15]. We specifically assessed if engaging in foraging in a navigational 3D large-scale environment, which intrinsically entailed higher costs in terms of distance travelled, induced people to deploy more cognitively costly strategies.

It has been previously emphasised that foraging in large-scale environments may require different psychological processes than those assumed in visual search [10]. Nonetheless, studies of search and foraging in large and small-scale environments often use different tasks [11]. By implementing similar tasks in 2D and 3D, in this study we were able to systematically manipulate individual variables to identify key sources of spontaneous changes in switching strategies in these environments.

Kristjánsson et al. [29] implemented foraging tasks, which they had previously developed [9,18], in a virtual reality environment where a static observer was required to shoot, as in a Space Invaders game, targets presented in a three-dimensional display. The main motivation for the study was to determine if previous results, obtained with a two-dimensional display presented on an iPad, could be generalised to a three-dimensional environment, which was considered more naturalistic. Their main comparisons were between conditions with targets defined by individual features or a conjunction of features. The authors replicated most of the findings of Kristjánsson et al. [9,18]. However, in the 2020 study [29] the authors found a less clear-cut difference between feature and conjunction conditions than they had previously observed using a 2D display. Kristjánsson et al. [29] suggested that this may have been because foraging in three-dimensional environments induces people to switch more even when holding conjunction templates. Thus, reducing the difference between the frequency of switches previously observed in these two conditions. They also compared conditions where the targets were static and conditions – referred to as dynamic – where the targets moved around. However, this latter manipulation produced only marginal effects. The study by Kristjánsson et al. [29] used 3D displays in visual foraging and provided insights on the switching behaviour of observers shooting at stimuli from a fixed location.

Our motivation for using virtual reality was different. We tested the hypothesis that the requirements of foraging in a navigational environment would provide an incentive to use more cognitively effortful strategies, characterised by a higher frequency of switches between target categories. Thus, the type of environment (3D navigational versus a 2D bird’s eye view) was one of our main manipulations of Experiment 1.

Experiment 1 confirmed that foraging in a 3D virtual environment induced a higher frequency of switching than foraging in a small-scale 2D environment. Participants likely switched more in the 3D large-scale environment to maximise how many selections they can make while avoiding travel costs. This is because selecting all the stimuli of one target type (e.g., red spheres), and subsequently selecting all the stimuli of the other target type (e.g., green spheres) would normally require more travelling. The fact that such a relationship existed was confirmed by a negative relationship between the frequency of switching and distance travelled to complete a trial in both 3D conditions of Experiment 1. Based on these findings, we suggest that participants switched more in the 3D large-scale environment to improve foraging efficiency, similarly to what would be expected from optimality models [47], even if this implied the use of more cognitively costly strategies. The fact that people switched more between targets in the large-scale 3D environment, which was also the environment that generated more errors and trial terminations, suggests that it was not difficulty to drive a reduction in the number of switches.

There are however other differences between foraging in 2D and 3D search spaces, in addition to the time and movement distance that they implicitly entail. Perhaps the most intuitive of these differences is the dynamic change in viewpoint implied by moving within an immersive environment. In fact, the distinction between the highly costly foraging behaviour of mobile searching predators compared to the lower costs implied by stationary foragers has long been recognised within the behavioural ecology and optimal foraging literature [48,49].

Thus, given the complex pattern of variables that may have had a role in producing the effects of our first experiment, we carried out additional experiments to clarify the respective role of speed and stimulus distance, as well as that of feature and conjunction foraging, in separate experiments carried out in 2D and 3D respectively. Experiments 2a and 2b were performed to investigate the effects of distance and speed in a 2D environment. In Experiment 2a we manipulated distance between stimuli and found that an increase in distance resulted in a significant increase in the number of switches performed by the participants. In Experiment 2b, the speed at which the cursor could move was manipulated while the distance between stimuli was kept constant. There, we observed that reducing movement speed also produced a significant increase in the number of switches performed by participants while foraging. These results reinforce the notion that there is a trade-off between cognitive costs and costs in distance travelled and time even when foraging takes place within a two-dimensional display. Travelling distance and time are often used as proxies of energetic costs and ultimately fitness in the optimal foraging literature [5]. Our participants changed search strategies as a function of these task demands. Strategies featuring a higher number of switches were used in presence of increased costs in terms of distance travelled and time spent searching. By default, people seem to prefer strategies that do not require many switches (and presumably bursts of concentration to hold multiple working memory templates for sustained periods; [18]). However, people choose more cognitively costly strategies, when not doing so would lead to increased movement costs. It must be stressed that these conclusions are drawn based on analyses of correct trials only (since errors resulted in the termination of the trial). However, more trials were terminated in those conditions where we observed fewer switches. Taking the number of trials terminated in any condition as an indication of the difficulty of the condition, we cannot exclude a contribution of the relative difficulty of dense conditions, perhaps induced by crowding, in the reduction of switches. The same explanations could not hold for Experiment 2b as the distance between items was kept constant in all conditions there. Yet, more switches were observed in slow conditions, which were those where more errors were observed. The significant negative correlation between frequency of switching and distance travelled observed in most conditions of Experiment 2b also supports the interpretation of an increased number of switches being motivated by an attempt to reduce movement length.

We carried out a further set of two experiments (Experiments 3a and 3b) to assess in a 3D environment: 1) the extent to which stimulus distance and movement speed affected the number of switches between target categories; and 2) the relative weight of the two factors (stimulus distance and speed of movement) in 3D. Thus, Experiments 3a and 3b were carried out in 3D large-scale rather than in the 2D small-scale environment used for Experiment 2a and 2b. Moreover, in a variation of the experimental design used for Experiments 2a and 2b, Experiments 3a and 3b featured both a 2 (stimulus distance) x 2 (speed of movement) design, but in a foraging task with stimuli defined by single features or conjunction of features, respectively. Interestingly, we found that in both Experiments 3a and 3b, only the manipulation of distance had a significant effect on the frequency of switches. A larger stimulus distance induced switching at a higher frequency than a shorter stimulus distance. However, in Experiments 3a and 3b dense conditions were also those where more trials were removed. As such, a contributing effect of difficulty, or crowding, in the reduction of switch frequency there cannot be excluded. There was also a significant negative relationship between switching frequency and movement distance covered to complete a trial in most conditions of experiment 3a and 3b. Overall, the results suggest that, at least for the parameters of distance and speed used in Experiments 3a and 3b, distance influenced the frequency of switches mostly.

We envisage two alternative reasons for this. The first is that whereas search time is dependent on the distance travelled, travelling speed does not affect distance. This asymmetrical causal relationship between spatial distance and time may contribute to the higher weight assigned to distance in the choice of foraging strategies.

Alternatively, it is possible that a precedence effect explains the higher saliency of distance perception over travelling speed. In fact, in our experimental set-up, it was possible for participants to apprehend the relative distance of the stimuli at trial outset (see Fig 8 and 10). By contrast, the speed of movement could only be noted after participants started moving. This precedence in the possible appreciation of stimulus distance at the beginning of a trial may have made people attend preferentially to this variable and bias their strategies for the rest of the trial. Further studies would be needed to determine which of such possibilities is more likely to have occurred. Nonetheless, irrespective of the specific reasons why distance travelled should have more weight than movement speed in determining people’s foraging choices, our results highlight the importance of separating the influence of these two variables, which are often intertangled in foraging studies.

Overall, the results of the set of experiments presented here provide clear evidence that people can dynamically change frequency of switching between stimulus categories in visual foraging, depending on the specific task demands of the type of foraging they engage with. This is a change that has been considered related to the flexible use of strategies requiring more or less cognitive effort to sustain multiple working memory templates for prolonged periods of time during foraging. We show that foraging in different environments, each representative of diverse but ecologically plausible search spaces, determines whether people engage in more or less cognitively demanding strategies. This supports models of visual foraging that envisage the flexible use of working memory templates [9,18]. Moreover, it clarifies which variables affect human strategy choice in a task domain that is so pervasive in human cognition.
